# Associations between XRCC2 rs3218536 and ERCC2 rs13181 polymorphisms and ovarian cancer

**DOI:** 10.18632/oncotarget.13361

**Published:** 2016-11-15

**Authors:** Wei Zhang, Zhifen Zhang

**Affiliations:** ^1^ Department of Gynecology, Nanjing Medical University, Affiliated Hangzhou Hospital (Hangzhou First People's Hospital), 310006, Hangzhou, China

**Keywords:** ERCC2, XRCC2, ovarian cancer, meta-analysis, single nucleotide polymorphism

## Abstract

Recent studies explored XRCC2 rs3218536 and ERCC2 rs13181 polymorphisms and ovarian cancer (OC) risk. However, the association between these two single nucleotide polymorphisms and OC risk remains conflicting. Thus, we conducted a comprehensive systematic review and meta-analysis to investigate the association. We searched the databases of PubMed, and Embase. Pooled odds ratios (ORs) and 95% confidence intervals (CIs) were calculated by using fixed-effect or random-effect models. 15 case-control studies published in 11 papers including 4,757 cases and 8,431 controls were included in this meta-analysis. No associations were obtained between XRCC2 rs3218536 and ERCC2 rs13181 polymorphisms and OC risk. Stratification analyses of Hardy–Weinberg equilibrium status indicated that rs3218536 polymorphism was associated with the decreased risk of OC when in analysis of combined HWE positive studies. In conclusion, this meta-analysis indicates that XRCC2 rs3218536 and ERCC2 rs13181 polymorphisms may not be associated with the risk of OC.

## INTRODUCTION

Ovarian cancer (OC), leading cause of gynaecologic cancer death, is the second most common gynaecologic cancer [1]. OC is mainly classified into four subtypes: serous, endometrioid, mucinous, and clear cell. Most of malignant OCs are of epithelial origin [2]. To date, the pathogenesis of OC still remains unclear. Multiple factors including age, family history of OC, gravidity, genetic and other environmental factors might be account for the etiology of OC [3, 4]. The known ovarian cancer susceptibility genes explain nearly 40% of the excess familial risk of OC [5].

DNA repair system takes part in maintaining the genomic integrity. The repair process usually contains two stages: the excision of lesion and the repair synthesis [6]. The repair system acts by mismatch repair (MMR), nucleotide excision repair (NER), and base-excision repair (BER). The repair by recombination removes a host of serious DNA lesions, encompassing double-stranded breaks (DSBs). These breaks induce a loss of some chromosomes and causes translocation of genetic material between them. Studies provided evidence to support the association between DSBs repair gene variants and ovarian cancer [7, 8]. The X-ray cross-complementing (XRCC) genes are DNA repair genes. These genes are associated with the DNA damage processing and genetic stability [9]. Studies have demonstrated that XRCC2 gene participates in homologous recombination of DNA [10]. Excision repair cross-complimentary group 2 (ERCC2), called xeroderma pigmentosum complementation group D (XPD), is involved in the NER pathway. ERCC2 removes certain DNA cross-links, ultraviolet photolesions, and bulky chemical adducts [11]. We hypothesized that those DNA repair genes (XRCC2 and ERCC2) are significantly associated with the risk of OC.

A number of studies [6, 9, 11–18] investigated the relationship between XRCC2 rs3218536 and ERCC2 rs13181 polymorphisms and OC susceptibility, but with conflicting results. Thus, we conducted a comprehensive meta-analysis to explore the possible association between XRCC2 rs3218536 and ERCC2 rs13181 polymorphisms and OC risk.

## RESULTS

### Characteristics of the included studies

**Figure 1:**
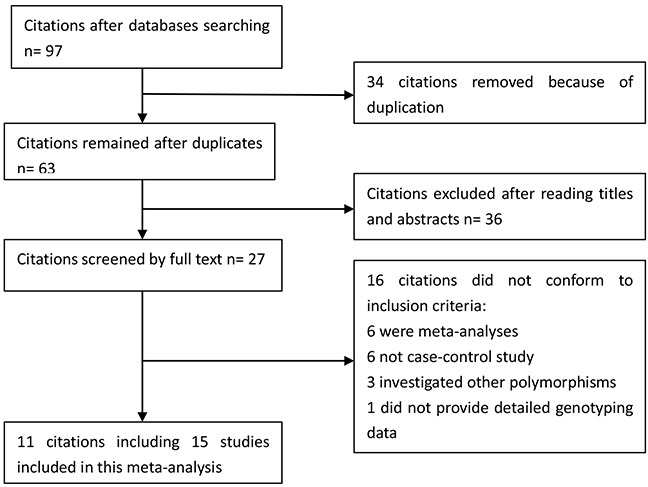
Selection for eligible citations included in this meta-analysis

We yielded 97 citations after initial searching. 34 citations were removed after removing duplicates. After screening the titles and abstracts, 36 citations were excluded. 27 citations were selected for further full text review. 16 citations were excluded: 6 were meta-analyses [19–24]; 6 not case-control studies [25–30]; 3 investigated other polymorphisms [14, 31, 32]; 1 did not provide detailed genotyping data [33]. We finally included 11 eligible citations [6, 9, 11–18] including 15 studies (4,757 cases and 8,431 controls) in this meta-analysis. Selection for eligible studies included in this meta-analysis was presented in Figure 1. The characteristics of included studies are summarized in Table 1. The Newcastle-Ottawa Scale (NOS) scores of all included studies ranged from 5 to 7 scores.

**Table 1:** Characteristics of included studies

Author and year	Country	Genotype method	SOC	Ethnicity	Case			Control			HWE	NOS
XRCC2 rs3218536					Arg/Arg	Arg/His	His/His	Arg/Arg	Arg/His	His/His		
Auranen_2005a	UK	TaqMan	PB	Caucasian	629	98	2	704	129	9	Y	6
Auranen_2005b	Denmark	PCR	PB	Caucasian	260	54	1	331	68	5	Y	6
Auranen_2005c	USA	PCR	PB	Caucasian	238	31	0	484	75	2	Y	7
Auranen_2005d	UK	PCR	PB	Caucasian	251	23	1	1538	267	6	Y	6
Webb_2005a	Australia	PCR-RFLP	HB	Caucasian	364	63	3	802	140	8	Y	5
Webb_2005b	Australia	PCR-RFLP	HB	Mixed	87	5	2	150	16	2	Y	7
Beesley_2007	Australia	MALDI-TOF	PB	Caucasian	799	117	7	696	115	7	Y	6
Mohamed_2013	Egypt	PCR	HB	Caucasian	6	58	36	16	60	24	N	6
Michalska_2016	Poland	PCR-RFLP	PB	Caucasian	120	80	500	180	400	120	N	7
**ERCC2 rs13181**					Lys/Lys	Lys/Gln	Gln/Gln	Lys/Lys	Lys/Gln	Gln/Gln		
Costa_2007	Portugal	PCR-RFLP	HB	Caucasian	55	49	22	95	95	12	Y	7
Bernard-Gallon_2008	France	TaqMan	HB	Caucasian	1	31	19	119	446	430	Y	5
Jakubowska_2010	Poland	PCR	HB	Caucasian	58	65	22	100	123	57	Y	6
Mohamed_2013	Egypt	PCR	HB	Caucasian	32	54	14	55	35	10	Y	7
Monteiro_2014	Brazil	PCR-RFLP	HB	Caucasian	33	36	1	37	30	3	Y	6
Michalska_2015	Poland	PCR-RFLP	PB	Caucasian	62	64	304	96	240	94	Y	6

SOC, source of controls; PB, population-based controls; HB, hospital-based controls; HWE, Hardy–Weinberg equilibrium; NOS, Newcastle-Ottawa Scale

### Quantitative synthesis

**Figure 2:**
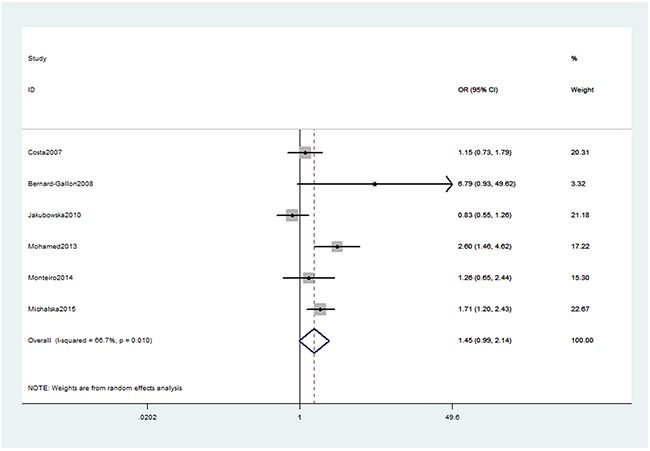
Forest plot shows odds ratio for the associations between ERCC2 rs13181 polymorphism and OC risk (Dominant model)

**Figure 3:**
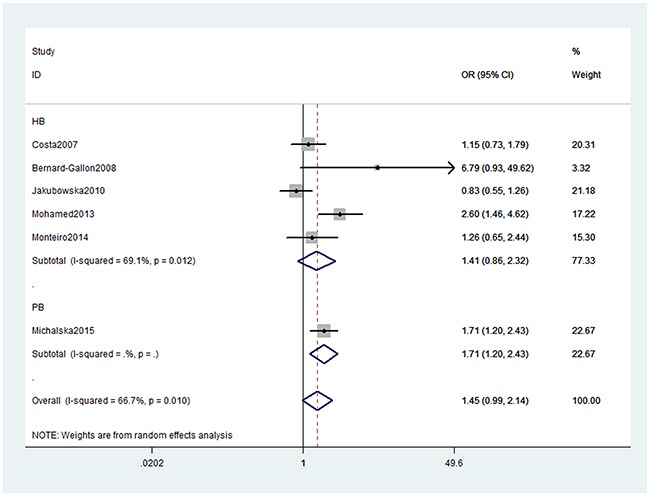
Stratification analyses by source of control between ERCC2 rs13181 polymorphism and OC risk (Dominant model)

As presented in Table 2, we obtained no significant association between XRCC2 rs3218536 or ERCC2 rs13181 (dominant: OR, 1.45; 95% CI, 0.99–2.14, *P* = 0.058, Figure 2) polymorphisms and the risk of OC. Stratification analyses were conducted according to HWE status, ethnicity and source of control (SOC). Our data indicated that XRCC2 rs3218536 polymorphism was significantly associated with a decreased risk of OC among HWE positive studies (Table 3). Regarding stratification analysis by SOC, we detected ERCC2 rs13181 polymorphism increased the risk of OC in population-based study (dominant model, Figure 3), while no association was found in both population-based and hospital-based studies. No significant association was obtained about rs3218536 polymorphism when performing stratification analyses by ethnicity.

**Table 2:** Meta-analysis of association between ERCC2 rs13181, XRCC2 rs3218536 polymorphisms and OC risk

Genetic models	OR(95%CI)	*P*-value	*P* for heterogeneity	I^2^ (%)	Model
**rs13181**					
Allele	1.45(0.83,2.54)	0.188	< 0.001	93.8	Random
Dominant	1.45(0.99,2.14)	0.058	0.010	66.7	Random
Recessive	1.56(0.49,4.93)	0.447	< 0.001	95.0	Random
**rs3218536**					
Allele	1.05(0.61,1.82)	0.852	< 0.001	96.4	Random
Dominant	0.96(0.74,1.24)	0.759	< 0.001	75.0	Random
Recessive	1.11(0.34,3.63)	0.862	< 0.001	91.7	Random

**Table 3:** Summary of the subgroup analyses in this meta-analysis

Comparison	Category	Category	Studies	OR (95% CI)	*P*-value
**rs3218536**					
Allele	SOC	PB	6	1.04(0.49,2.19)	0.926
		HB	3	1.13(0.77,1.66)	0.535
	HWE	Positive	7	0.84(0.74,0.95)	0.006
		Negative	2	2.57(1.03,6.38)	0.042
	Ethnicity	Caucasian	8	1.08(0.61,1.94)	0.783
		Asian	1	0.79(0.35,1.78)	0.577
Dominant	SOC	PB	6	0.91(0.67,1.24)	0.556
		HB	3	1.17(0.59,2.33)	0.649
	HWE	Positive	7	0.83(0.73,0.95)	0.007
		Negative	2	1.74(1.36,2.24)	<0.001
	Ethnicity	Caucasian	8	0.98(0.75,1.28)	0.887
		Asian	1	0.67(0.27,1.67)	0.390
Recessive	SOC	PB	6	0.92(0.15,5.80)	0.932
		HB	3	1.57(0.92,2.69)	0.098
	HWE	Positive	7	0.69(0.37,1.26)	0.225
		Negative	2	4.74(0.73,30.96)	0.104
	Ethnicity	Caucasian	8	1.05(0.29,3.73)	0.943
		Asian	1	1.80(0.25,13.02)	0.558
**rs13181**					
Allele	SOC	HB	5	1.19(0.89,1.59)	0.248
		PB	1	3.61(2.93,4.45)	<0.001
Dominant	SOC	HB	5	1.41(0.86,2.32)	0.168
		PB	1	1.71(1.20,2.43)	0.003
Recessive	SOC	HB	5	1.13(0.58,2.19)	0.726
		PB	1	8.62(6.33,11.75)	<0.001

**Figure 4:**
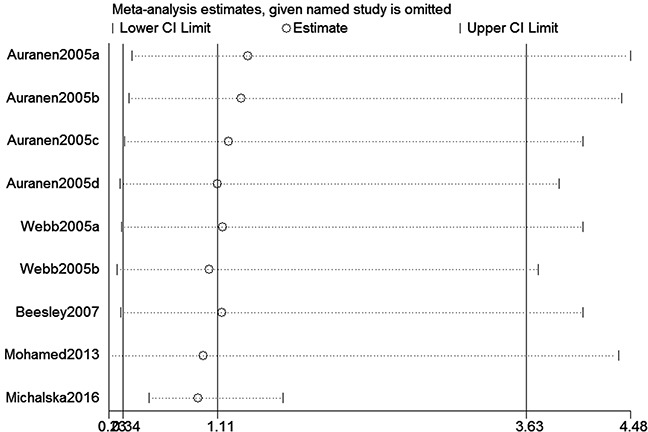
Sensitivity analysis about XRCC2 rs3218536 polymorphism and OC risk (Recessive model)

**Figure 5:**
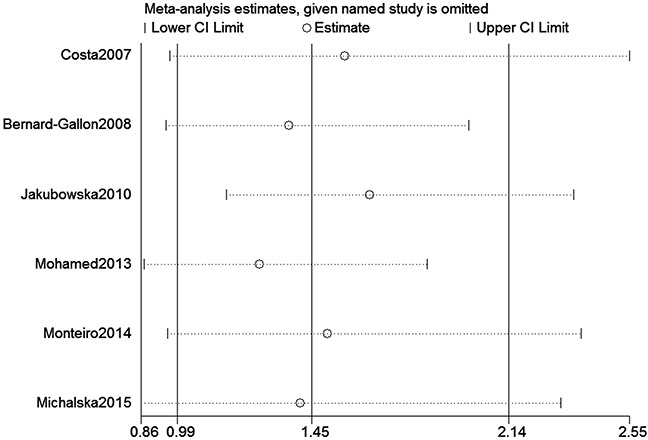
Sensitivity analysis about ERCC2 rs13181 polymorphism and OC risk (Dominant model)

We assessed sensitivity by omitting each study once at a time in every genetic model for XRCC2 rs3218536 or ERCC2 rs13181 polymorphisms. The pooled ORs for the effects about these two polymorphisms indicated that our data about the two SNPs were stable and trustworthy (rs3218536: recessive model, Figure 4; rs13181: dominant model, Figure 5). Begg's test was used to evaluate the publication bias of this meta-analysis (rs3218536, allele: *P* = 0.764, dominant: *P* = 1.000, and recessive: *P* = 0.532; rs13181, allele: *P* = 0.573, dominant: *P* = 0.348, and recessive: *P* = 0.851). Our data revealed that there was no obvious publication bias for the two SNPs. Due to significant between-study heterogeneity among some genetic models, we conductedmeta-regression to explore whether ethnicity, HWE status and SOCwere the resources of heterogeneity. However, our data suggested that ethnicity, HWE status and SOC did not seem to be responsible for the heterogeneity (data not shown).

## DISCUSSION

DNA repair systems are important for protecting against mutations and are necessary for maintaining the integrity of the genome. Many identified DNA repair genes are recognized to have genetic variations in humans [34]. DNA repair gene polymorphisms may alter the protein function. They can also cause reduction in DNA repair capacity, which may result in genetic instability and carcinogenesis [35, 36]. DNA damage influences mitosis and the isolation of chromosomes, which can be solved by homologous recombination repair (HRR) [37]. HRR is a pivotal pathway to repair the DSBs and maintain the genetic stability [38]. XRCC2 is involved in the HRR pathway and associated with DNA DSB repair and genomic stability [38, 39]. ERCC2 is one of seven nucleotide excision repair enzymes. ERCC2 could cause Xeroderma Pigmentosum when mutated in germ line. ERCC2 is involved in DNA repair, specifically in nucleotide excision repair. It functions in various types of DNA lesions [12]. Both XRCC2 and ERCC2 are identified as DNA repair genes.

A host of studies [6, 9, 11–18] have explored the associations between XRCC2 rs3218536 or ERCC2 rs13181 gene polymorphisms and OC risk. But they provided inconsistent results. These studies were conflicting and inconclusive may due to different ethnic populations, clinical heterogeneity, and small sample sizes. As a result, we conducted a meta-analysis to investigate the association. We found no evidence for an association with XRCC2 rs3218536 or ERCC2 rs13181 polymorphism. Stratification analyses of HWE status revealed that XRCC2 rs3218536 polymorphism was significantly associated with a decreased risk of OC in analysis of HWE positive studies. Studies conform to HWE, indicating control subjects were representative of the general population. Studies with deviation from HWE are prone to false positive results [40]. Further investigations are urgent to confirm the findings of stratification analyses.

Previous meta-analysis [20, 23] demonstrated the association with XRCC2 rs3218536 polymorphism, but with contradictory conclusions. Shi et al. found rs3218536 polymorphism reduced the risk of OC [23], while Zhai et al. indicated this SNP increased the risk of ovarian cancer [20]. However, our data suggested that this SNP was not associated with the risk of OC. Compared with previous meta-analysis, this meta-analysis included a new Polish study containing 700 cases and 700 controls. It is well recognized that the association between SNPs in genes with diseases is greatly affected by the number of subjects. Our study has larger sample size, indicating that our data are more robust. Stratification analyses of rs3218536 polymorphism by ethnicity suggested that no association was obtained with Caucasian population or mixed population.

To seek the sources of high heterogeneity in this meta-analysis, we conducted meta-regression analysis, stratification analyses, and sensitivity analysis. Meta-regression analysis of ethnicity, HWE status and SOC was conducted. Our data confirmed that ethnicity, HWE status and SOC were not the sources of heterogeneity. Sensitivity analysis about XRCC2 rs3218536 polymorphism indicated the Polish study [6] may be the source of heterogeneity. We found the heterogeneity reduced substantially in three genetic models (Allele, I^2^ = 48.9%; Dominant, I^2^ = 42.0%); Recessive, I^2^ = 19.6%) when we excluded this Polish study. The reasons of high heterogeneity may due to different ethnic populations, clinical heterogeneity, and small sample sizes.

However, potential limitations should be addressed in this meta-analysis. First, due to limited data, we could not perform further stratification analyses of other populations, such as Asians. Second, our results were based on unadjusted estimates for confounding factors, which might influence the final findings. Third, we could not assess potential gene-gene and gene-environment interactions. Fourth, the sample sizes of subgroup analysis are limited. These stratification analyses were based on small numbers and any association is likely to be due to chance. Fifth, high heterogeneity existed in some genetic models of this meta-analysis.

In summary, this meta-analysis suggests that XRCC2 rs3218536 and ERCC2 rs13181 polymorphisms may not be associated with OC susceptibility. Stratification analysis indicates that XRCC2 rs3218536 polymorphism was significantly associated with a decreased risk of OC when in analysis of HWE positive studies. Further studies are necessary to validate whether these two SNPs is associated with OC susceptibility in other ethnic groups.

## MATERIALS AND METHODS

### Literature search and inclusion criteria

We systematically searched the PubMed, and Embase to identify studies through September 13, 2016. The following search terms were used: “ovarian cancer,” “ovarian neoplasm,” “ovarian carcinoma,” “ERCC2,” “XPD,” “XRCC2” and “polymorphism”. Other potential omitted studies were identified by hand screening. The inclusion criteria of studies were as following: (1) studies that evaluated the association between XRCC2 rs3218536 and ERCC2 rs13181 polymorphisms and OC risk, (2) study provided sufficient data to calculate the odds ratios (ORs) and 95% confidence intervals (CIs), and (3) case-control study.

### Data extraction and quality assessment

Data was extracted from all eligible studies by two authors. The extracted information from all eligible studies including: name of first author, publication year, country, ethnicity, source of control, and genotype numbers of cases and controls. Two authors independently conducted the extraction of data. We assessed the study quality according to the NOS [41]. All disagreements were resolved by discussion until reaching consent.

### Statistical analysis

The crude ORs and 95%CIs were used to assess the strength of associations between XRCC2 rs3218536 and ERCC2 rs13181 polymorphisms and OC risk. Stratification analysis was carried out by HWE status, ethnicity and SOC. When a Q test indicated *P* < 0.1 or I^2^ > 50% indicated heterogeneity across studies, a random-effect model was used. Otherwise, the fixed-effects model was applied [42]. Pooled ORs were calculated for allele model, dominant model, and recessive model. We performed leave-one-out sensitivity analysis to evaluate the stability of the overall results. We assessed the departure from the HWE in the controls using Pearson's χ2 test. Begger's linear regression test was used to detect the potential publication bias [43]. Meta-regression analysis of ethnicity, HWE status and SOC was performed to seek the main sources of the heterogeneity. All statistical analyses were performed using the Stata 11.0 software (STATA Corporation, College Station, TX, USA).

## Abbreviations

OC: ovarian cancer
DSB: double-stranded break
XRCC: X-ray cross-complementing
ERCC2: Excision repair cross-complimentary group 2
MMR: mismatch repair
NER: nucleotide excision repair
BER: base-excision repair
CI: confidence interval
OR: odds ratio
NOS: Newcastle-Ottawa Scale
HWE: Hardy–Weinberg equilibrium
SNP: single nucleotide polymorphism

## References

[1] Pearce CL, Near AM, Van Den Berg DJ, Ramus SJ, Gentry-Maharaj A, Menon U, Gayther SA, Anderson AR, Edlund CK, Wu AH, Chen X, Beesley J, Webb PM, Holt SK, Chen C, Doherty JA (2009). Validating genetic risk associations for ovarian cancer through the international Ovarian Cancer Association Consortium. British journal of cancer.

[2] Slotman BJ, Rao BR (1988). Ovarian cancer (review). Etiology, diagnosis, prognosis, surgery, radiotherapy, chemotherapy and endocrine therapy. Anticancer research.

[3] Romero I, Bast RC (2012). Minireview: human ovarian cancer: biology, current management, and paths to personalizing therapy. Endocrinology.

[4] Murdoch WJ, Martinchick JF (2004). Oxidative damage to DNA of ovarian surface epithelial cells affected by ovulation: carcinogenic implication and chemoprevention. Experimental biology and medicine.

[5] Antoniou AC, Easton DF (2006). Risk prediction models for familial breast cancer. Future oncology.

[6] Michalska MM, Samulak D, Romanowicz H, Jablonski F, Smolarz B (2016). Association between single nucleotide polymorphisms (SNPs) of XRCC2 and XRCC3 homologous recombination repair genes and ovarian cancer in Polish women. Experimental and molecular pathology.

[7] Yuan C, Liu X, Yan S, Wang C, Kong B (2014). Analyzing association of the XRCC3 gene polymorphism with ovarian cancer risk. BioMed research international.

[8] Liang H, Li Y, Luo RY, Shen FJ (2014). An increased risk of ovarian cancer associated with polymorphism in BRCC5 gene in Caucasian populations. Tumour biology : the journal of the International Society for Oncodevelopmental Biology and Medicine.

[9] Mohamed FZ, Hussien YM, AlBakry MM, Mohamed RH, Said NM (2013). Role of DNA repair and cell cycle control genes in ovarian cancer susceptibility. Molecular biology reports.

[10] Silva SN, Tomar M, Paulo C, Gomes BC, Azevedo AP, Teixeira V, Pina JE, Rueff J, Gaspar JF (2010). Breast cancer risk and common single nucleotide polymorphisms in homologous recombination DNA repair pathway genes XRCC2, XRCC3, NBS1 and RAD51. Cancer epidemiology.

[11] Michalska MM, Samulak D, Romanowicz H, Sobkowski M, Smolarz B (2015). An Association between Single Nucleotide Polymorphisms of Lys751Gln ERCC2 Gene and Ovarian Cancer in Polish Women. Advances in medicine.

[12] Monteiro MS, DB Vilas Boas, Gigliotti CB, Salvadori DM (2014). Association among XRCC1, XRCC3, and BLHX gene polymorphisms and chromosome instability in lymphocytes from patients with endometriosis and ovarian cancer. Genetics and molecular research : GMR.

[13] Beesley J, Jordan SJ, Spurdle AB, Song H, Ramus SJ, Kjaer SK, Hogdall E, DiCioccio RA, McGuire V, Whittemore AS, Gayther SA, Pharoah PD, Webb PM (2007). Association between single-nucleotide polymorphisms in hormone metabolism and DNA repair genes and epithelial ovarian cancer: results from two Australian studies and an additional validation set. Cancer epidemiology, biomarkers & prevention : a publication of the American Association for Cancer Research, cosponsored by the American Society of Preventive Oncology.

[14] Jakubowska A, Gronwald J, Menkiszak J, Gorski B, Huzarski T, Byrski T, Toloczko-Grabarek A, Gilbert M, Edler L, Zapatka M, Eils R, Lubinski J, Scott RJ, Hamann U (2010). BRCA1-associated breast and ovarian cancer risks in Poland: no association with commonly studied polymorphisms. Breast cancer research and treatment.

[15] Bernard-Gallon D, Bosviel R, Delort L, Fontana L, Chamoux A, Rabiau N, Kwiatkowski F, Chalabi N, Satih S, Bignon YJ (2008). DNA repair gene ERCC2 polymorphisms and associations with breast and ovarian cancer risk. Molecular cancer.

[16] Webb PM, Hopper JL, Newman B, Chen X, Kelemen L, Giles GG, Southey MC, Chenevix-Trench G, Spurdle AB (2005). Double-strand break repair gene polymorphisms and risk of breast or ovarian cancer. Cancer epidemiology, biomarkers & prevention : a publication of the American Association for Cancer Research, cosponsored by the American Society of Preventive Oncology.

[17] Costa S, Pinto D, Pereira D, Vasconcelos A, Afonso-Lopes C, Osorio T, Lopes C, Medeiros R (2007). Importance of xeroderma pigmentosum group D polymorphisms in susceptibility to ovarian cancer. Cancer letters.

[18] Auranen A, Song H, Waterfall C, Dicioccio RA, Kuschel B, Kjaer SK, Hogdall E, Hogdall C, Stratton J, Whittemore AS, Easton DF, Ponder BA, Novik KL, Dunning AM, Gayther S, Pharoah PD (2005). Polymorphisms in DNA repair genes and epithelial ovarian cancer risk. International journal of cancer.

[19] Zhang Y, Wang H, Peng Y, Liu Y, Xiong T, Xue P, Du L (2014). The Arg188His polymorphism in the XRCC2 gene and the risk of cancer. Tumour biology : the journal of the International Society for Oncodevelopmental Biology and Medicine.

[20] Zhai M, Wang Y, Jiang MF (2015). Arg188His polymorphism in the XRCC2 gene and the risk of ovarian cancer: a meta-analysis. Genetics and molecular research : GMR.

[21] Michalska MM, Samulak D, Smolarz B (2014). An association between the -41657 C/T polymorphism of X-ray repair cross-complementing 2 (XRCC2) gene and ovarian cancer. Medical oncology.

[22] Wu KG, He XF, Li YH, Xie WB, Huang X (2014). Association between the XPD/ERCC2 Lys751Gln polymorphism and risk of cancer: evidence from 224 case-control studies. Tumour biology : the journal of the International Society for Oncodevelopmental Biology and Medicine.

[23] Shi S, Qin L, Tian M, Xie M, Li X, Qi C, Yi X (2014). The effect of RAD51 135 G>C and XRCC2 G>A (rs3218536) polymorphisms on ovarian cancer risk among Caucasians: a meta-analysis. Tumour biology : the journal of the International Society for Oncodevelopmental Biology and Medicine.

[24] He Y, Zhang Y, Jin C, Deng X, Wei M, Wu Q, Yang T, Zhou Y, Wang Z (2014). Impact of XRCC2 Arg188His polymorphism on cancer susceptibility: a meta-analysis. PloS one.

[25] White KL, Vierkant RA, Fogarty ZC, Charbonneau B, Block MS, Pharoah PD, Chenevix-Trench G, AACSg for, Rossing MA, Cramer DW, Pearce CL, Schildkraut JM, Menon U, Kjaer SK, Levine DA, Gronwald J (2013). Analysis of over 10,000 Cases finds no association between previously reported candidate polymorphisms and ovarian cancer outcome. Cancer epidemiology, biomarkers & prevention : a publication of the American Association for Cancer Research, cosponsored by the American Society of Preventive Oncology.

[26] Kang S, Sun HY, Zhou RM, Wang N, Hu P, Li Y (2013). DNA repair gene associated with clinical outcome of epithelial ovarian cancer treated with platinum-based chemotherapy. Asian Pacific journal of cancer prevention : APJCP.

[27] Lambrechts S, Lambrechts D, Despierre E, Van Nieuwenhuysen E, Smeets D, Debruyne PR, Renard V, Vroman P, Luyten D, Neven P, Amant F, Leunen K, Vergote I, Belgian and Luxembourg Gynaecological Oncology G (2015). Genetic variability in drug transport, metabolism or DNA repair affecting toxicity of chemotherapy in ovarian cancer. BMC pharmacology & toxicology.

[28] Rump A, Benet-Pages A, Schubert S, Kuhlmann JD, Janavicius R, Machackova E, Foretova L, Kleibl Z, Lhota F, Zemankova P, Betcheva-Krajcir E, Mackenroth L, Hackmann K, Lehmann J, Nissen A, DiDonato N (2016). Identification and Functional Testing of ERCC2 Mutations in a Multi-national Cohort of Patients with Familial Breast- and Ovarian Cancer. PLoS genetics.

[29] Peethambaram P, Fridley BL, Vierkant RA, Larson MC, Kalli KR, Elliott EA, Oberg AL, White KL, Rider DN, Keeney GL, Cunningham JM, Hartmann LC, Goode EL (2011). Polymorphisms in ABCB1 and ERCC2 associated with ovarian cancer outcome. International journal of molecular epidemiology and genetics.

[30] Fleming ND, Agadjanian H, Nassanian H, Miller CW, Orsulic S, Karlan BY, Walsh CS (2012). Xeroderma pigmentosum complementation group C single-nucleotide polymorphisms in the nucleotide excision repair pathway correlate with prolonged progression-free survival in advanced ovarian cancer. Cancer.

[31] C Leigh Pearce, Near AM, Butler JL, Van Den Berg D, Bretsky P, Conti DV, Stram DO, Pike MC, Hirschhorn JN, Wu AH (2008). Comprehensive evaluation of ESR2 variation and ovarian cancer risk. Cancer epidemiology, biomarkers & prevention : a publication of the American Association for Cancer Research, cosponsored by the American Society of Preventive Oncology.

[32] Sellers TA, Schildkraut JM, Pankratz VS, Vierkant RA, Fredericksen ZS, Olson JE, Cunningham J, Taylor W, Liebow M, McPherson C, Hartmann LC, Pal T, Adjei AA (2005). Estrogen bioactivation, genetic polymorphisms, and ovarian cancer. Cancer epidemiology, biomarkers & prevention : a publication of the American Association for Cancer Research, cosponsored by the American Society of Preventive Oncology.

[33] Khokhrin DV, Khrunin AV, Moiseev AA, Gorbunov VA, Limborskaia SA (2012). [Association of polymorphisms in glutathione-S-transferase and DNA repair genes with ovarian cancer risk in the Russian population]. Genetika.

[34] Debniak T, Scott RJ, Huzarski T, Byrski T, Masojc B, van de Wetering T, Serrano-Fernandez P, Gorski B, Cybulski C, Gronwald J, Debniak B, Maleszka R, Kladny J, Bieniek A, Nagay L, Haus O (2006). XPD common variants and their association with melanoma and breast cancer risk. Breast cancer research and treatment.

[35] Berwick M, Vineis P (2000). Markers of DNA repair and susceptibility to cancer in humans: an epidemiologic review. Journal of the National Cancer Institute.

[36] de Boer JG (2002). Polymorphisms in DNA repair and environmental interactions. Mutation research.

[37] Thacker J (1999). A surfeit of RAD51-like genes?. Trends in genetics : TIG.

[38] Tambini CE, Spink KG, Ross CJ, Hill MA, Thacker J (2010). The importance of XRCC2 in RAD51-related DNA damage repair. DNA repair.

[39] Thacker J (2005). The RAD51 gene family, genetic instability and cancer. Cancer letters.

[40] Ziegler A, Van Steen K, Wellek S (2011). Investigating Hardy-Weinberg equilibrium in case-control or cohort studies or meta-analysis. Breast cancer research and treatment.

[41] Stang A (2010). Critical evaluation of the Newcastle-Ottawa scale for the assessment of the quality of nonrandomized studies in meta-analyses. European journal of epidemiology.

[42] Higgins JP, Thompson SG (2002). Quantifying heterogeneity in a meta-analysis. Statistics in medicine.

[43] Peters JL, Sutton AJ, Jones DR, Abrams KR, Rushton L (2006). Comparison of two methods to detect publication bias in meta-analysis. Jama.

